# Chronic exposure to polystyrene microplastics induced male reproductive toxicity and decreased testosterone levels via the LH-mediated LHR/cAMP/PKA/StAR pathway

**DOI:** 10.1186/s12989-022-00453-2

**Published:** 2022-02-17

**Authors:** Haibo Jin, Minghao Yan, Chun Pan, Zhenyu Liu, Xiaoxuan Sha, Chengyue Jiang, Luxi Li, Mengge Pan, Dongmei Li, Xiaodong Han, Jie Ding

**Affiliations:** 1 Immunology and Reproductive Biology Laboratory and State Key Laboratory of Analytical Chemistry for Life Science, Medical School, Nanjing University, Hankou Road 22, Nanjing, 210093 Jiangsu China; 2 Jiangsu Key Laboratory of Molecular Medicine, Nanjing University, Nanjing, 210093 China

**Keywords:** PS-MPs, Reproductive toxicity, Testosterone, LHR

## Abstract

**Background:**

Microplastics (MPs), which are smaller in size and difficult to degrade, can be easily ingested by marine life and enter mammals through the food chain. Our previous study demonstrated that following acute exposure to MPs, the serum testosterone content reduced and sperm quality declined, resulting in male reproductive dysfunction in mice. However, the toxic effect of long-term exposure to MPs at environmental exposure levels on the reproductive system of mammals remains unclear.

**Results:**

In vivo, mice were given drinking water containing 100 μg/L and 1000 μg/L polystyrene MPs (PS-MPs) with particle sizes of 0.5 μm, 4 μm, and 10 μm for 180 consecutive days. We observed alterations in testicular morphology and reductions in testosterone, LH and FSH contents in serum. In addition, the viability of sperm was declined and the rate of sperm abnormality was increased following exposure to PS-MPs. The expression of steroidogenic enzymes and StAR was downregulated in testis tissues. In vitro, we used primary Leydig cells to explore the underlying mechanism of the decrease in testosterone induced by PS-MPs. First, we discovered that PS-MPs attached to and became internalized by Leydig cells. And then we found that the contents of testosterone in the supernatant declined. Meanwhile, LHR, steroidogenic enzymes and StAR were downregulated with concentration-dependent on PS-MPs. We also confirmed that PS-MPs decreased StAR expression by inhibiting activation of the AC/cAMP/PKA pathway. Moreover, the overexpression of LHR alleviated the reduction in StAR and steroidogenic enzymes levels, and finally alleviated the reduction in testosterone induced by PS-MPs.

**Conclusions:**

PS-MPs exposure resulted in alterations in testicular histology, abnormal spermatogenesis, and interference of serum hormone secretion in mice. PS-MPs induced a reduction in testosterone level through downregulation of the LH-mediated LHR/cAMP/PKA/StAR pathway. In summary, our study showed that chronic exposure to PS-MPs resulted in toxicity of male reproduction under environmental exposure levels, and these potential risks may ring alarm bells of public health.

**Graphical abstract:**

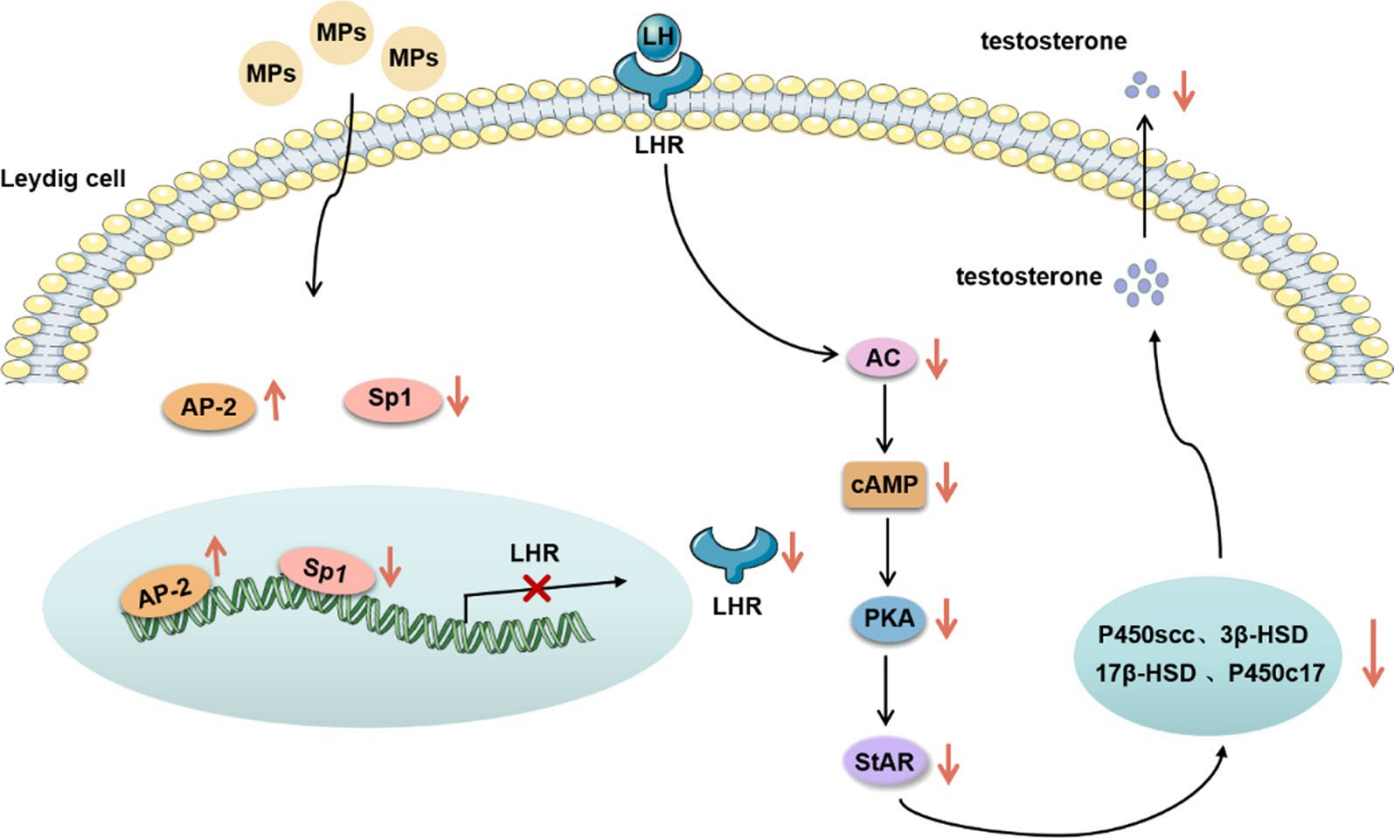

**Supplementary Information:**

The online version contains supplementary material available at 10.1186/s12989-022-00453-2.

## Background

The use of plastic products has been increasing for decades, leading to the serious pollution of plastic waste [1, 2]. After wave action, UV radiation, photodegradation, and biodegradation, plastic wastes are degraded into fragments and particles [3, 4]. Microplastics (MPs), with diameters less than 5 mm, have attracted broad attention [5, 6], because of their small size, stable chemical properties and difficulty in degradation [7]. MPs are widely distributed in water environments and terrestrial environments [8, 9]. MPs are mainly discharged directly into the water environments through sewage treatment plants [10]. Small plastic particles also could be spread further by landfills or other surface deposits, and resulting MPs sink in terrestrial environments [11]. The main components of MPs are polyethylene terephthalate (PET), polyethylene (PE), polystyrene (PS), polypropylene (PP), and polyvinyl chloride (PVC) in the environment [12, 13]. MPs may be accumulated in organisms to cause toxicity. Moreover, MPs can be easily transported into food chains and ultimately threaten human health [14, 15]. Therefore, special attention should be given to the toxicity of MPs.

Previous studies have demonstrated that MPs are harmful to multiple organs in aquatic organisms and mammals, such as the liver, kidney, gastrointestinal tract, and brain [16–25]. The reproductive system plays a crucial role in organisms. Reproductive toxicity of MPs has been found in aquatic organisms, such as Daphnia, Hydra attenuate [25], medaka fish [26], and oyster [27]. A few researchers demonstrated that MPs induced decreased sperm quality, disordered hormone levels, and oxidative stress in the testis following acute and short-term exposure [28–30]. However, the toxic effect of MPs chronic exposure on the reproductive system of mammals remains unclear.

Our previous study identified that following PS-MPs exposure for 28 days, testicular histology altered, serum testosterone levels decreased and sperm quality declined, resulting in male reproductive dysfunction in mice. Testosterone, the most important androgen in males, plays an important role in spermatogenesis. A decreased testosterone level is strongly correlated with functional damage in the testes and Leydig cells [31, 32]. Therefore, we hypothesized that PS-MPs induced male reproductive toxicity in mice by down-regulating testosterone levels. Nevertheless, the underlying mechanism of the decrease in testosterone levels caused by MPs remains unexplored. Testosterone is synthesized and secreted by Leydig cells [33]. LH, which is released by the anterior pituitary, binds to LH receptor (LHR) located on the membrane of Leydig cells, resulting in increased cAMP content. Then, protein kinase A (PKA) is activated and the levels of steroidogenic acute regulatory protein (StAR) and steroid synthase (P450scc, P450c17, 3β-HSD, 17β-HSD) are increased. Under the action of StAR, free cholesterol in the cytoplasm is transported from the outer membrane of mitochondria to the inner membrane. P450scc on the intima converts cholesterol into pregnenolone. Pregnenolone sequentially enters the endoplasmic reticulum, where it is converted to testosterone by 3β-HSD, P450c17, and 17β-HSD [34]. In this study, we focused on the effect of PS-MPs on testosterone synthesis in testicular Leydig cells.

In addition, previous studies reported that the toxicity of MPs in different tissues is closely related to their particle size. Deng et al. showed that 5 μm and 20 μm PS-MPs exposure induced disturbances in energy and lipid metabolism [20]. Jeong et al. demonstrated that 0.05 μm, 0.5 μm, and 6 μm PS-MPs treatment led to a reduction in the growth rate and fecundity, and pointed out that the toxicity of MPs was size-dependent and smaller microbeads were more toxic [35]. Meanwhile, some studies elucidated that the toxicity of MPs was concentration dependent [21, 23, 36]. Therefore, we sought to investigate the effects of the different sizes and concentrations of MPs on the male reproductive system in mice.

In the present study, we exposed mice to MPs of different particle sizes (0.5 μm, 4 μm and 10 μm) at different concentrations to investigate the male reproductive toxicity of MPs. Meanwhile, we used a primary Leydig cell model to explore the underlying mechanism of the decrease in testosterone levels induced by MPs. Our findings may provide novel insight into preventing the reproductive toxicity of MPs.

## Results

### Characterization of polystyrene microplastics (PS-MPs)

**Fig. 1:**
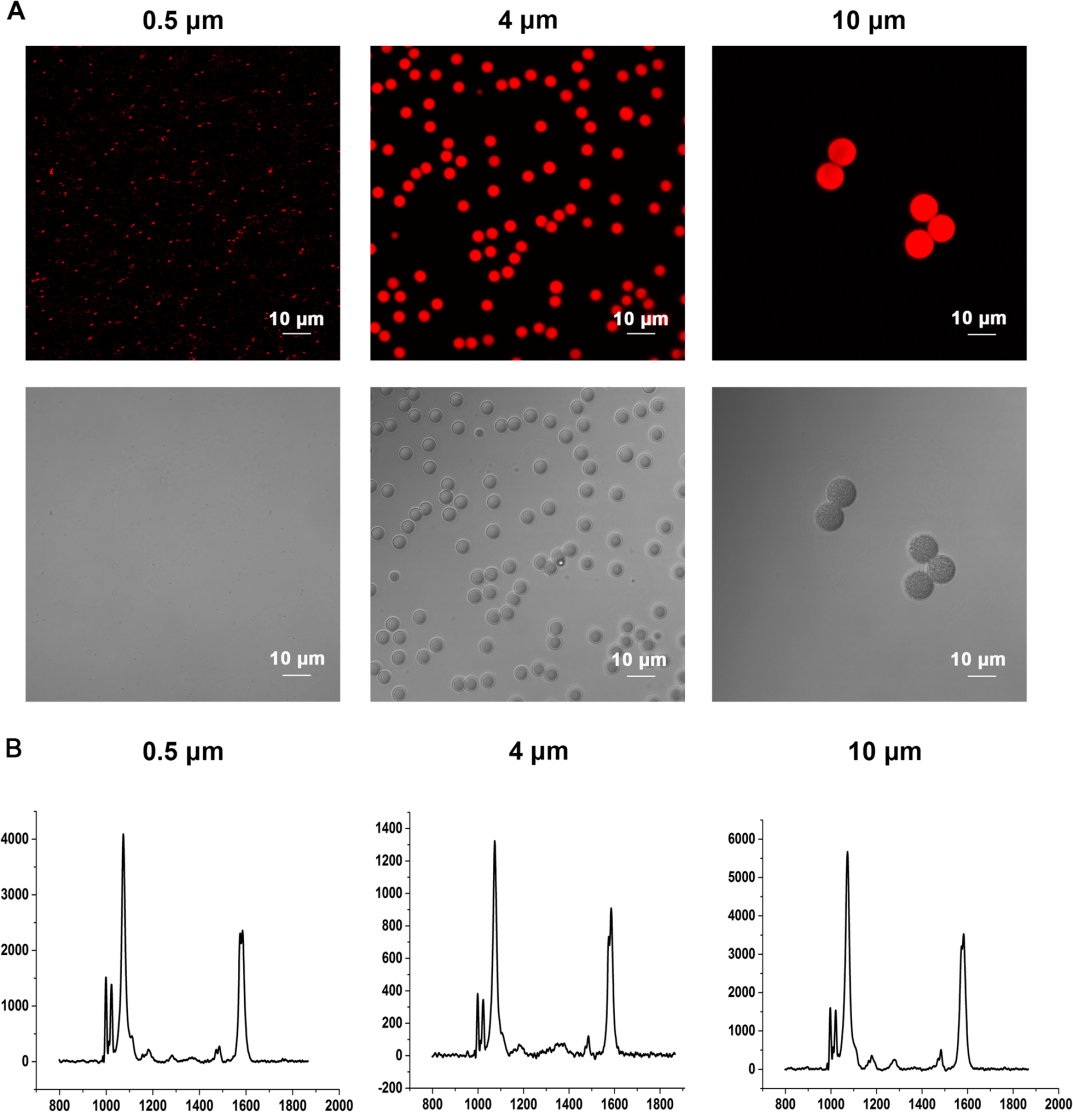
Characterization of polystyrene microplastics (PS-MPs). **A** Morphological detection of PS-MPs. Confocal imaging showing various sizes of PS-MPs. **B** Raman spectrum characterizations of PS-MPs

Confocal imaging was conducted to detect the morphology and sizes of PS-MPs used in this study. As shown in Fig. 1A, the MPs were spheres, and the sizes of MPs met the design requirements. According to Raman spectra analysis, the monomer of MPs was polystyrene (Fig. 1B). The zeta potential values were shown in Additional file 1: Table S1.

### Exposure to PS-MPs induced testicular tissue structure damage and sperm quality decrease in mice

**Fig. 2:**
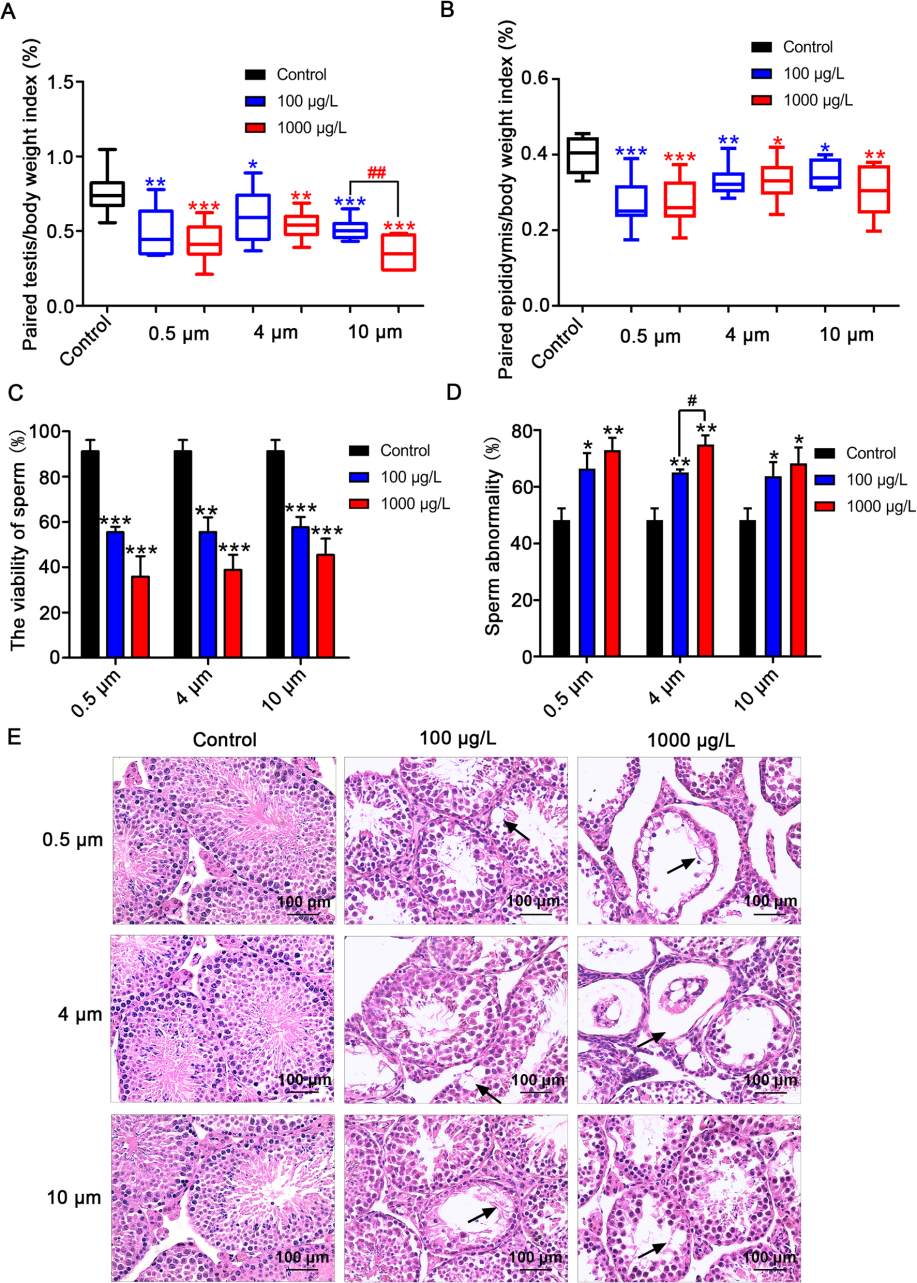
PS-MPs exposure induced reproductive toxicity in male mice. Mice were given drinking water containing different sizes of PS-MPs at 100 μg/L and 1000 μg/L for 180 continuous days. **A** The paired testis/body weight index was assessed (n = 15). **B** The paired epididymis/body weight index was analyzed (n = 15). **C** The viability of sperm was detected (n = 15). **D** The rate of sperm abnormality was calculated (n = 15). **E** The impact of PS-MPs on testis structures was examined by H&E staining. Arrows: abnormal structure. Data represent means ± SD. *P < 0.05, **P < 0.01, ***P < 0.001 vs. control. ^#^*P* < 0.05, ^##^*P* < 0.01 vs. 100 μg/L group

During the period of treatment, we monitored the body weights, consumption of food and water. The body weights and food consumption of mice were decreased (Additional file 1: Table S2). However, there was no remarkable change in water intake among the different groups. Compared with the control group, the testicular coefficient (Fig. 2A) and epididymal coefficient (Fig. 2B) decreased significantly in the 0.5 μm, 4 μm, and 10 μm groups. Meanwhile, the testicular coefficient of the 1000 μg/L PS-MPs with a diameter of 10 μm group decreased more obviously than that of the 100 μg/L group. Following exposure to PS-MPs for 180 days, we detected the viability and morphology of sperm. The results revealed that PS-MPs exposure decreased the viability of sperm (Fig. 2C) and increased the rate of sperm deformity (Fig. 2D). In addition, photomicrographs of testes in control and experimental mice were displayed in Fig. 2E. Control testicular sections presented with normal testicular architecture, intact epithelial membrane with normal spermatogenesis and lumen full of sperm. Conversely, degenerative, atrophied tubules with vacuolated tubular generation, derangement of cell layers, and abscission of spermatogenic cells were observed in the PS-MPs exposure groups. Moreover, exposure to higher concentrations of PS-MPs could cause more severe damage to testicular structures. Evaluation of morphometric parameters in testicular cross-sections of PS-MPs exposure groups showed significantly reduced tubular diameter (Additiona1 file 1: Fig. S1A), germinal cell thickness (Additiona1 file 1: Fig. S1B).

### Exposure to PS-MPs reduced the content of testosterone, LH and FSH in serum

**Fig. 3:**
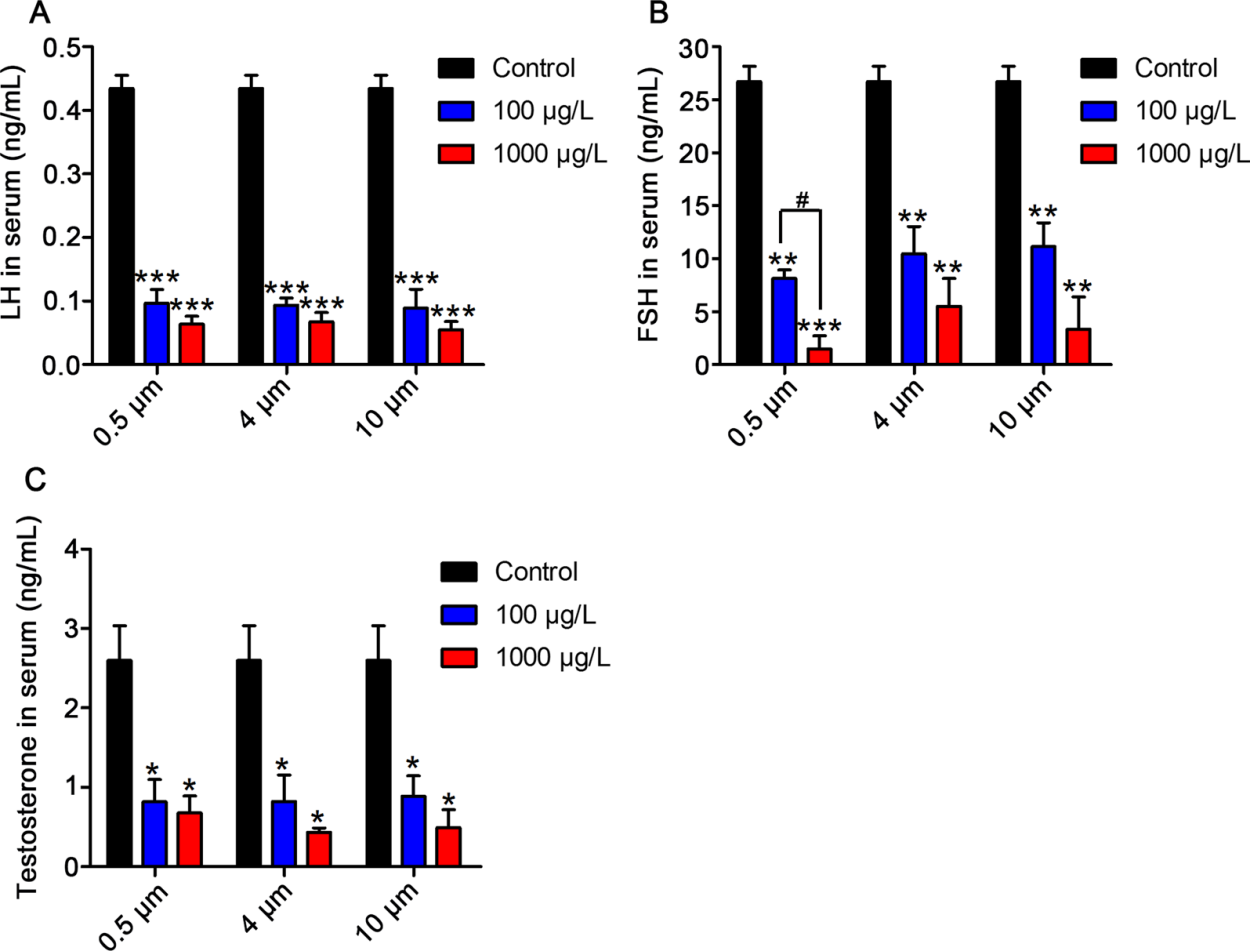
PS-MPs exposure decreased the content of testosterone, LH, and FSH in serum. The contents of LH (**A**), FSH (**B**), and testosterone (**C**) in serum were measured by ELISA. Results are expressed as means ± SD. *P < 0.05, **P < 0.01, ***P < 0.001 vs. control. ^#^*P* < 0.05 vs. 100 μg/L group

To explore the effects of PS-MPs exposure on the concentration of reproductive hormones, we detected the levels of testosterone, LH and FSH in serum. The results indicated that LH levels were apparently decreased in a dose-dependent manner after treatment with PS-MPs (Fig. 3A). As shown in Fig. 3B, the concentrations of FSH in serum exhibited an appreciable decrease in all treatment groups, and the decrease was most obvious in the 0.5 μm PS-MPs treatment group. Meanwhile, the concentrations of testosterone in serum were markedly decreased following exposure to various sizes of PS-MPs (Fig. 3C).

### PS-MPs downregulated the expression of steroidogenic enzymes and StAR in mice

**Fig. 4:**
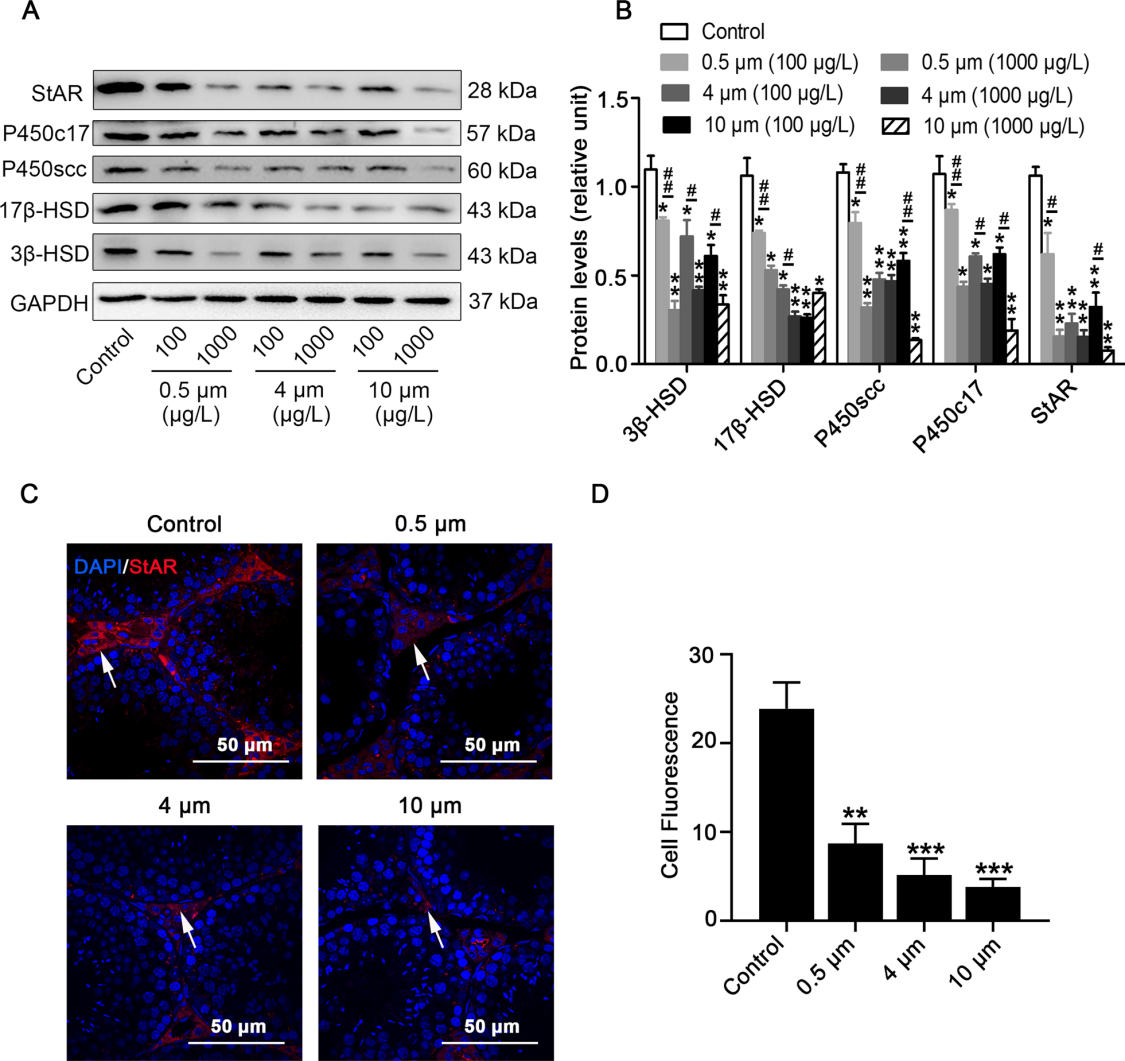
PS-MPs treatment downregulated the expression of steroidogenic enzymes and StAR in testes. Mice were given drinking water comprising PS-MPs of various sizes for 180 sustained days. **A**, **B** The expression of 3β-HSD, 17β-HSD, P450scc, P450c17, and StAR in testes was measured by western blotting. The expression levels were quantified with ImageJ (n = 3). Data are expressed as means ± SD. *P < 0.05, **P < 0.01 vs. control. ^#^*P* < 0.05, ^##^*P* < 0.01 vs. 100 μg/L group. **C**, **D** The expression of StAR in mouse testicular tissues of the control group and 1000 μg/L group was tested by immunofluorescence staining. Testicular tissues were stained with StAR (red) and DAPI (blue), arrows: positive expression (scale bar = 50 µm). The expression levels of StAR were quantified with ImageJ (n = 3). Data are expressed as means ± SD. **P < 0.01 vs. control. ^#^*P* < 0.05, ***P < 0.001 vs. control

3β-HSD is a marker for Leydig cells. Following exposure to PS-MPs, the number of 3β-HSD^+^ cells in testes was markedly decreased in comparison to the control group, as assessed by immunohistochemistry assay (Additiona1 file 1: Fig. S2). To assess whether PS-MPs may affect testosterone synthesis, we detected the levels of enzymes correlated with testosterone synthesis in testis tissues. The results showed that the expression of P450scc, P450c17, 3β-HSD, and 17β-HSD was downregulated in the testis tissues (Fig. 4A and B). For the same particle size of PS-MPs, the decrease was more obvious in the 1000 μg/L exposure group than in the 100 μg/L exposure group. In addition, we detected StAR in the testes of PS-MPs-treated mice by western blotting (Fig. 4A and B) and immunofluorescence analyses (Fig. 4C). StAR was only detected in the cells located in the testicular interstitium and not observed within the seminiferous tubules. StAR was apparently decreased in the PS-MPs-treated group compared with the control group (Fig. 4D).

### Entering of PS-MPs into Leydig cells in vitro

**Fig. 5:**
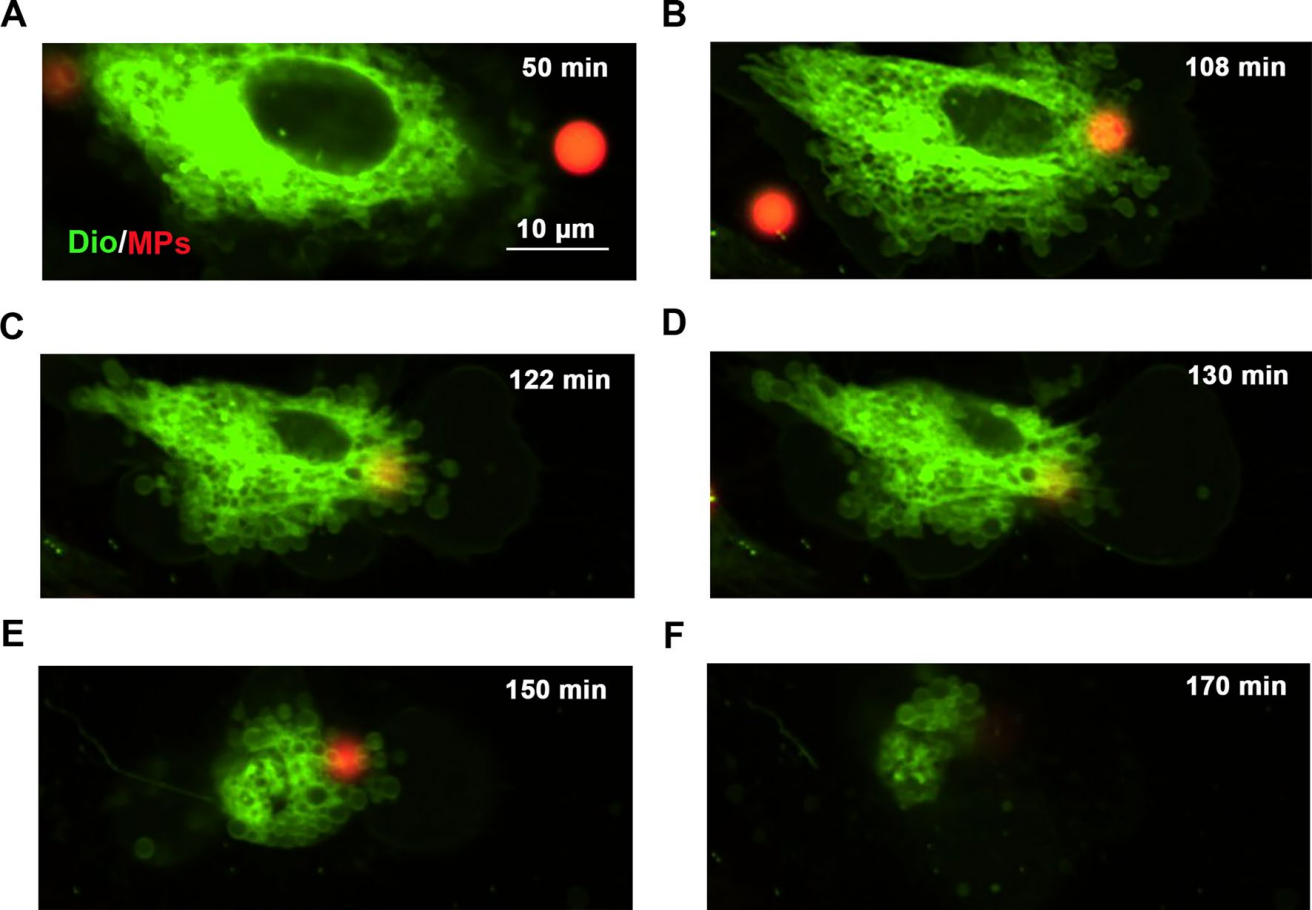
Images of particle-cell interactions of PS-MPs. Leydig cells were exposed to 4 μm PS-MPs. Spinning disc confocal images of the cells with fluorescently labeled filamentous actin (green) and PS-MPs (red) (scale bar = 10 μm). The pictures were captured at 50 min (**A**), 108 min (**B**), 122 min (**C**), 130 min (**D**), 150 min (**E**), and 170 min (**F**) to show obvious changes

Confocal imaging results revealed that PS-MPs attached to and became internalized by Leydig cells (Fig. 5). After PS-MPs were endocytosed, the Leydig cells became curled up and could not be restored to its original morphology.

### PS-MPs inhibited the expression of testosterone, steroidogenic enzymes and StAR in primary Leydig cells

**Fig. 6:**
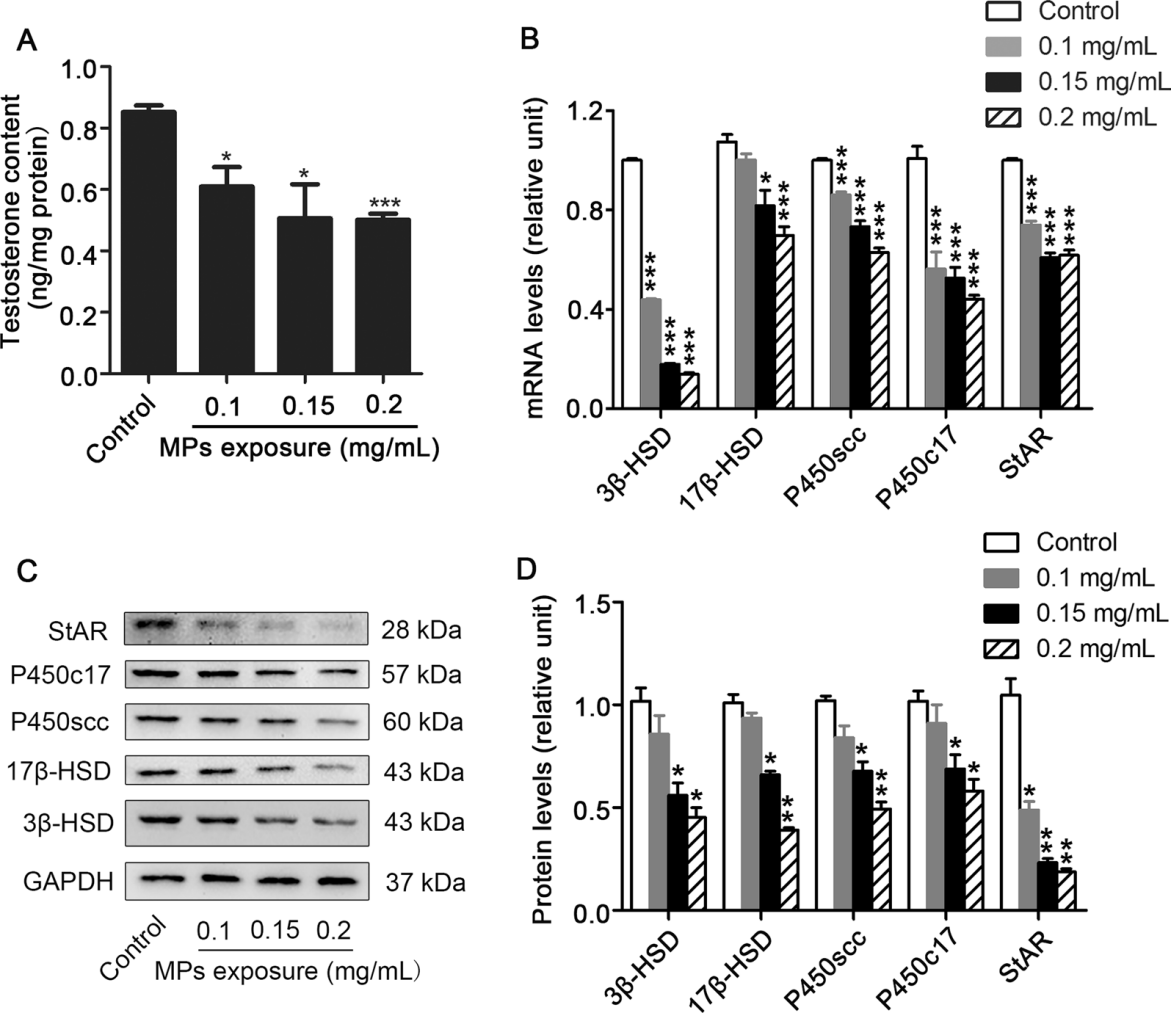
The expression of steroidogenic enzymes and StAR decreased in Leydig cells after PS-MPs treatment. Primary Leydig cells were exposed to 0.5 μm PS-MPs for 24 h at various concentrations as indicated. **A** The content of testosterone in supernatant was determined by ELISA assays (n = 3). **B** The mRNA expression levels of 3β-HSD, 17β-HSD, P450scc, P450c17, and StAR in Leydig cells after treatment with PS-MPs were determined by qRT-PCR (n = 3). **C**, **D** The expression of 3β-HSD, 17β-HSD, P450scc, and P450c17 in cells was measured by western blotting. The expression levels were quantified with ImageJ (n = 3). Data are expressed as means ± SD. *P < 0.05, **P < 0.01, ***P < 0.001 vs. control

Testosterone synthesis is related to steroid synthase in Leydig cells. We exposed Leydig cells to various concentrations of PS-MPs, and examined the concentrations of testosterone in the supernatant by ELISA. Figure 6A showed that the testosterone contents declined following treatment with PS-MPs. QRT-PCR results verified that the mRNA levels of the above enzymes were all decreased (Fig. 6B). Subsequently, we detected the levels of steroidogenic enzymes in Leydig cells. A concentration-dependent decreasing trend of P450scc, P450c17, 3β-HSD, and 17β-HSD was observed following exposure to PS-MPs in Leydig cells (Fig. 6C and D). Meanwhile, the expression of StAR in Leydig cells was decreased remarkably with increasing PS-MPs concentrations (Fig. 6B, C and D).

### PS-MPs exposure inhibited the activation of the AC/cAMP/PKA pathway in primary Leydig cells

**Fig. 7:**
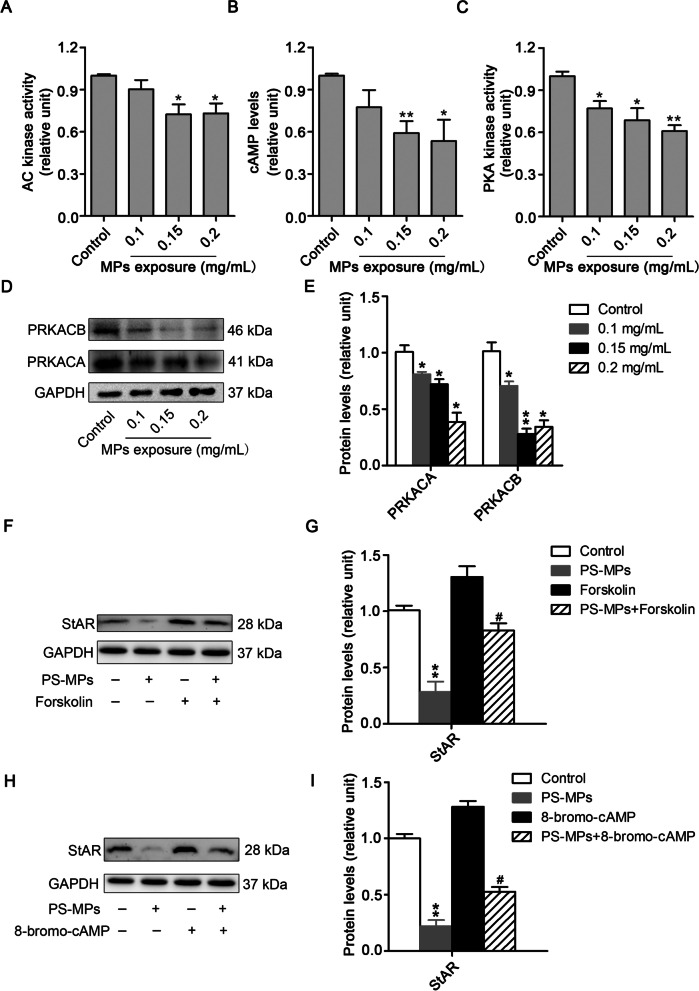
PS-MPs decreased the level of StAR by inhibiting the AC/cAMP/PKA pathway. Primary Leydig cells were exposed to various concentrations of 0.5 μm PS-MPs for 24 h. **A** AC kinase activity was measured by an AC activity assay kit (n = 3). Results are expressed as means ± SD. *P < 0.05, **P < 0.01 vs. control. **B** The content of cAMP was detected by ELISA (n = 3). Results are expressed as means ± SD. *P < 0.05, **P < 0.01 vs. control. **C** PKA kinase activity was examined by a PKA activity assay kit (n = 3). Results are expressed as means ± SD. *P < 0.05, **P < 0.01 vs. control. (D, E) The expression of PRKACA and PRKACB in cells was analyzed by western blotting. The expression levels were quantified with ImageJ and expressed as means ± SD (n = 3). Results are expressed as means ± SD. *P < 0.05, **P < 0.01 vs. control. (F, G) Leydig cells were incubated with 0.2 mg/mL PS-MPs for 1 h and then cultured in serum-free medium in the presence of 20 mM forskolin for another 24 h. The expression of StAR in cells was analyzed by western blotting. The expression levels were quantified with ImageJ and expressed as means ± SD (n = 3). Results are expressed as means ± SD. **P < 0.01 vs. control; ^#^*P* < 0.05 vs. forskolin treatment group. (H, I) Leydig cells were incubated with 0.2 mg/mL PS-MPs for 1 h and then cultured in serum-free medium in the presence of 0.1 mM 8-bromo-cAMP for another 24 h. The expression of StAR in cells was analyzed by western blotting. The expression levels were quantified with ImageJ and expressed as means ± SD (n = 3). Results are expressed as means ± SD. **P < 0.01 vs. control; ^#^*P* < 0.05 vs. 8-bromo-cAMP treatment group

**Fig. 8:**
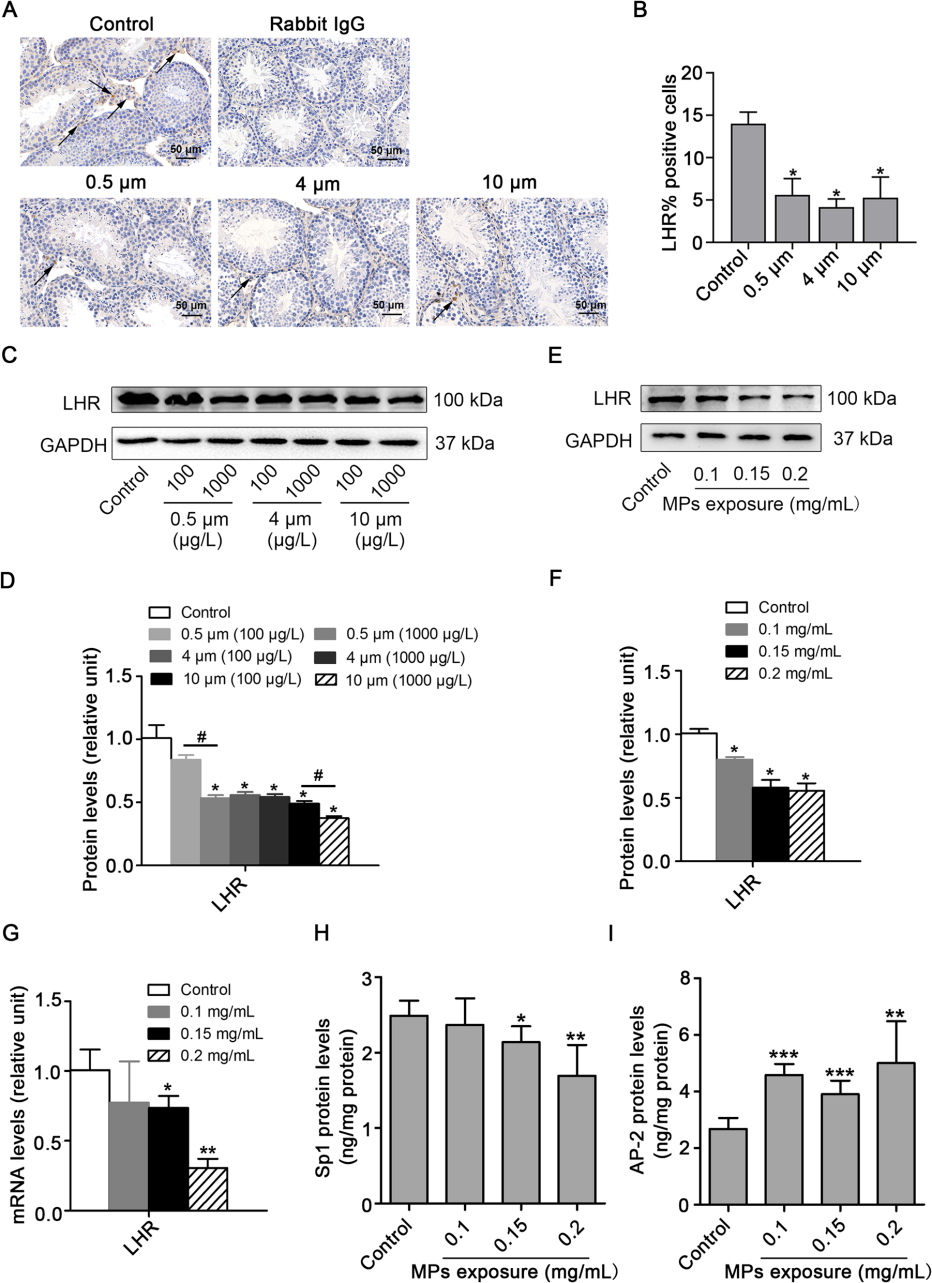
PS-MPs induced a decrease in testosterone levels by reducing LHR levels. **A**, **B** Mice were given drinking water containing different sizes of PS-MPs for 180 continuous days. The testis tissue sections were prepared for immunohistochemical staining with LHR antibody, arrows: positive expression (scale bar = 50 µm, n = 3). Percent of positivity was calculated based on the percentage of LHR positive cells out of the total number of cells in an image (*P < 0.05 vs. the control). **C**, **D** Mice were given drinking water containing various sizes of PS-MPs for 180 continuous days. The expression of LHR in testes was measured by western blotting. The expression levels were quantified with ImageJ and expressed as means ± SD (n = 3; *P < 0.05 vs. control; ^#^*P* < 0.05 vs. 100 μg/L group). **E**, **F** Primary Leydig cells were exposed to 0.1, 0.15, and 0.2 mg/mL PS-MPs with a diameter of 0.5 μm for 24 h. The expression of LHR in cells was analyzed by western blotting. The expression levels were quantified with ImageJ and expressed as means ± SD (n = 3; *P < 0.05 vs. control). **G** The mRNA expression levels of LHR in Leydig cells after treatment with PS-MPs were determined by qRT-PCR (n = 3; *P < 0.05, **P < 0.01 vs. control). (H, I) The contents of Sp1 and AP-2 were detected by ELISA (n = 3; *P < 0.05, **P < 0.01, ***P < 0.001 vs. control)

To explore the role of the AC/cAMP/PKA pathway in the expression of StAR, we first observed that AC kinase activity was reduced in PS-MPs-treated Leydig cells (Fig. 7A). ELISA analysis revealed that the content of cAMP declined following treatment with PS-MPs (Fig. 7B). Moreover, PKA kinase activity was dampened with the dose of PS-MPs-exposure (Fig. 7C), and PKA catalytic subunits (PRKACA and PRKACB) decreased with the concentration of PS-MPs-exposure (Fig. 7D and E). These results elucidated that the AC/cAMP/PKA pathway was involved in the inhibition of StAR expression by PS-MPs. To examine this assumption, we used 20 mM forskolin (cAMP agonist) and 0.1 mM 8-bromo-cAMP (PKA agonist) to treat cells. The results indicated that the reduction in StAR levels caused by PS-MPs was alleviated after treatment with forskolin (Fig. 7F and G) and 8-bromo-cAMP (Fig. 7H and I). Collectively, these results implied that PS-MPs decreased StAR expression by inhibiting activation of the AC/cAMP/PKA pathway.

### Exposure to PS-MPs induced a decrease in LHR levels in testes and primary Leydig cells

In general, LH binds to LHR, which is located on the membrane of Leydig cells, resulting in activation of the AC/cAMP/PKA pathway. To explore the specific reason for AC/cAMP/PKA pathway inhibition induced by PS-MPs, we detected the expression of LHR in testes (Fig. 8 A, B, C and D) and Leydig cells (Fig. 8E and F). Immunohistochemistry and Western blotting analysis revealed that PS-MPs induced a reduction in LHR levels. QRT-PCR results showed that the mRNA level of LHR was decreased (Fig. 8G). ELISA results revealed that the transcriptional enhancer Sp1 was downregulated and the suppressor AP-2 was upregulated (Fig. 8H and I).

### Regulation of LHR affected the levels of StAR, steroidogenic enzymes and testosterone in PS-MPs-exposed primary Leydig cells

**Fig. 9:**
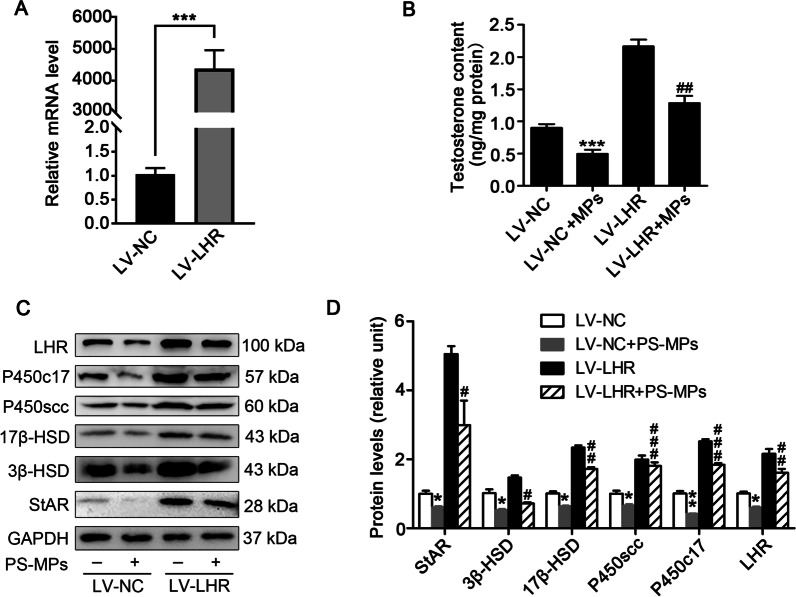
Overexpression of LHR alleviated the reduction in StAR, steroidogenic enzymes and testosterone levels induced by PS-MPs treatment in primary Leydig cells. **A** Primary cells were infected with LHR or empty vector with lentivirus for 72 h. The mRNA levels of LHR were tested by qRT-PCR (n = 3; ***P < 0.001 vs. LV-NC group). **B** Primary cells were infected with LHR or empty vector with lentivirus for 72 h. Then, the cells were exposed to 0.5 μm PS-MPs at a concentration of 0.2 mg/mL for 24 h. The testosterone content in the supernatant was examined by ELISA (n = 3; ***P < 0.001 vs. LV-NC group; ^##^*P* < 0.01 vs. LV-NC + PS-MPs group). **C**, **D** Primary cells were infected with LHR or empty vector with lentivirus for 72 h. Then, the cells were treated with 0.5 μm PS-MPs with a concentration at 0.2 mg/mL for 24 h. The expression of 3β-HSD, 17β-HSD, P450scc, P450c17, StAR, and LHR in cells was measured by western blotting. The expression levels were quantified with ImageJ (n = 3; *P < 0.05, **P < 0.01 vs. LV-NC group; ^#^*P* < 0.05, ^##^*P* < 0.01, ^###^*P* < 0.01 vs. LV-NC + PS-MPs group)

To determine the role of LHR in the decrease in testosterone levels induced by PS-MPs in Leydig cells, the cells were transfected with LHR by lentivirus. QRT-PCR and western blotting analysis suggested that the level of LHR in the Leydig cells transfected with LHR by lentivirus was overexpressed (Fig. 9A and C). The levels of testosterone and enzymes involved in testosterone synthesis under basal conditions or treatment with PS-MPs were checked in Leydig cells infected with LHR or empty vector by lentivirus. As shown in Fig. 9B and C, the overexpression of LHR alleviated the reduction of testosterone and steroidogenic enzymes levels induced by PS-MPs.

## Discussion

Recent studies have demonstrated that microplastics (MPs) have adverse effect on male reproduction and sperm quality, suggesting MPs are threats for male fertility [37]. Hou et al. demonstrated that polystyrene MPs (PS-MPs) decreased sperm quality in mice by activating the Nrf2/HO-1/NF-κB pathway [28]. Xie et al. revealed that PS-MPs exposure activated the p38 MAPK signaling pathway and induced oxidative stress in mouse testes, resulting in reproductive toxicity in mice [29]. Fatemeh Amereh et al. indicated that the concentrations of testosterone, LH and FSH in the serum of male rats were reduced. Meanwhile, DNA damage and alterations in sperm morphology were obvious [30]. This is the first study to explore the reproductive effects of long-term MPs-exposure at environmental levels. In this study, the choice of MPs concentrations (100 μg/L and 1000 μg/L) based on previous studies and was close to those found in natural contaminated areas [19, 23, 38]. We planned to try to explore the relationship between the degree of toxicity and particle size of MPs, so we chose three sizes (0.5 μm, 4 μm, 10 μm) of MPs to conduct the animal experiments in vivo. We discovered that PS-MPs treatment induced alterations of testicular histology and abnormal spermatogenesis in vivo. The secretion of LH and FSH is stimulated by gonadotropin releasing hormone derived from hypothalamus. In present study, we demonstrated that the concentration of testosterone, LH and FSH in serum were decreased, which suggested that PS-MPs might affect the hypothalamic–pituitary–gonadal (HPT) axis and hormone balance. Normally, when testosterone biosynthesis is sufficiently suppressed, it can directly stimulate the secretion of LH by adenohypophysis and GnRH by hypothalamus through a negative feedback mechanism. However, the feedback mechanism only occurs when the testosterone level reaches a certain concentration. The level of hormones in the blood is in a dynamic process. The reduction in the LH serum concentration may be directly responsible for the reduction in testosterone production by Leydig cells in PS-MPs-exposed mice. Moreover, we found concentration-dependent decreases among sperm abnormalities in the 4 μm group, FSH levels in the 0.5 μm group, and several steroidogenic enzymes levels in the three sizes groups. However, we could not find a significant difference among the three sizes in indexes related to reproductive ability. These results were similar to those of some studies that found PS-MPs of 0.5 and 50 μm were no difference to induce gut microbiota dysbiosis [38].

Testosterone plays a crucial role in the process of spermatogenesis and male reproductive system [32]. Testosterone is indispensable for pivotal processes during spermatogenesis, such as maintenance of the BTB [39–41], meiosis [42], Sertoli-spermatid adhesion [43] and sperm release [44]. The synthesis and secretion of testosterone are mainly regulated by LH-dependent signaling pathways in Leydig cells [33, 34]. In this study, primary Leydig cells were used to discuss the mechanisms of the severely decreased testosterone levels caused by PS-MPs treatment. The doses of PS-MPs used in vitro experiments were selected for the purpose of building a cell model of PS-MPs-induced testosterone-reduction for subsequent studies on the mechanism. In our previous study, we demonstrated that three particle sizes (0.5 μm, 4 μm, and 10 μm) of fluorescent PS-MPs could enter Leydig cells [45]. For best observation, we chose 4 μm PS-MPs for living cell imaging, that could show very clearly process of PS-MPs were internalized by Leydig cells. The results were similar to those of a previous study that demonstrated that environmentally exposed MPs particles were internalized significantly into macrophages [46].

In addition, we confirmed that PS-MPs treatment affected the levels of steroidogenic enzymes in Leydig cells. StAR is an important regulator of steroid hormone synthesis. Meanwhile, we discovered that following exposure to PS-MPs, the expression of StAR was obviously suppressed. Many factors affect the expression of StAR, such as the cAMP/PKA signaling pathway, protein kinase C, and regulatory factors [47–49]. However, the cAMP/PKA signaling pathway was the main factor affecting the expression of StAR. In our results, inhibition of the cAMP/PKA signaling pathway was observed, and the decrease in StAR expression induced by PS-MPs was alleviated following treatment with a cAMP agonist and PKA agonist. In general, the cAMP/PKA signaling pathway was activated when LH bound to LHR. Meanwhile, we found that PS-MPs triggered a decrease in LHR protein levels, and overexpression of LHR alleviated the reduction in testosterone levels induced by PS-MPs. In conclusion, MPs reduced testosterone levels by regulating the testosterone synthesis pathway which was dependent on LH, and LHR was the critical initiator.

In this study, we first demonstrated that long-term exposure to PS-MPs at environmental pollution concentrations caused damage to testicular tissue structure, decreased sperm quality and decreased testosterone levels, resulting in male reproductive toxicity in mice. In addition, we showed that the decrease in testosterone levels induced by PS-MPs was achieved by inhibiting the LH-mediated LHR/cAMP/PKA/StAR pathway. Moreover, LHR may play a critical role in the decrease of testosterone levels induced by PS-MPs.

## Conclusions

In this study, alteration of testicular histology, abnormal spermatogenesis, and reduction in testosterone, LH and FSH content in serum were observed in mice following exposure to 0.5 μm, 4 μm, and 10 μm PS-MPs in environmentally polluted concentrations for 180 days. The synthesis of testosterone is regulated by LH, and LHR is critical to the synthesis of testosterone in Leydig cells. We observed that PS-MPs triggered a decrease in LHR level in testes and Leydig cells, which further decreased the cAMP content and PKA activity, leading to decreased expression of StAR and steroidogenic enzymes. Taken together, PS-MPs induced a reduction in testosterone level through downregulation of the LH-mediated LHR/cAMP/PKA/StAR pathway, resulting in male reproductive disorder. Our findings may provide new perspectives for understanding the reproductive toxicity of PS-MPs in mammals.

## Materials and methods

### Test materials

Fluorescent polystyrene microplastics (PS-MPs) (10 mg/mL) of different sizes (0.5 μm, 4 μm, 10 μm) were purchased from Tianjin Baseline ChromTech Research Centre (Tianjin,

China). Fetal bovine serum (FBS) was obtained from ExCell Bio (Shanghai, China) and Dulbecco’s modified Eagle’s medium (DMEM)-F12 was obtained from Gibco (Grand Island, NY). Penicillin–streptomycin were gained from Sigma-Aldrich (St.Louis, MO).

### Characterization of PS-MPs

The 5 μL PS-MPs (10 mg/mL) was added to 2 mL ddH_2_O. The morphology of PS-MPs was detected using an FV10i microscope (Olympus, Japan). The monomer of MPs was examined using a Raman microscope (InVia; Renishaw, Inc., Illinois, USA). The instrument was set as follows: laser with 785 nm edge; grating of 1200 l/mm (633/780); center of spectrum range of 1150 Raman shift/cm − 1; exposure time of 1 s; laser power of 100%.

### Animals and treatment

Specific pathogen-free (SPF) male BALB/c mice aged six weeks were obtained from the Medical School of Yangzhou University (Yangzhou, China). Mice were divided into 7 groups with 15 mice in each group. Mice in each group were randomly placed in three cages (n = 5/cage). Mice in the PS-MPs exposure group were given drinking water containing 100 μg/L and 1000 μg/L PS-MPs with sizes of 0.5 μm, 4 μm, and 10 μm for 180 continuous days. For the control group, mice were provided blank water without PS-MPs. After the exposure period, mice were anesthetized to gain blood and testes. All experimental protocols were approved by the Animal Care and Use Committee of Nanjing University according to animal protocol number SYXK (Su) 2009-0017.

### Tissue collection

After 180 days of continuous exposure to PS-MPs, retroorbital plexus blood was collected under anesthesia. The blood was standing at room temperature for 30 min and centrifuged at 3000 rpm for 15 min. Blood serum were collected and stored at − 80° C. Mice were anesthetized to death to obtain testes for later analysis. A portion of the fresh testis tissues was quickly frozen with liquid nitrogen and stored at − 80° C, and the other portion of the testis tissues was placed in 4% paraformaldehyde for paraffin embedding.

### Cell culture and PS-MPs exposure

Primary Leydig cells were isolated from 3-week-old BALB/c male mouse testes, as described previously [50]. Cells were cultured in DMEM-F12 (Gibco, Grand Island, NY) supplemented with 10% FBS (ExCell Bio, Shanghai, China), 1% penicillin–streptomycin (Sigma-Aldrich, St.Louis, MO), and 0.1% human chorionic gonadotropin (hCG) (Thermo). After adherence for 48 h, cells were cultured in DMEM-F12 containing various concentrations (0.1, 0.15, and 0.2 mg/ml) of 0.5 μm PS-MPs for 24 h. In addition, cells were cultured in DMEM-F12 with 4 μm PS-MPs for living cell imaging detection.

### Agonist treatment

Cells were cultured in DMEM-F12 supplemented with 10% FBS for 96 h and then cultured in serum-free medium in the presence of 0.1% hCG and 0.2 mg/mL PS-MPs for 1 h. Subsequently, 20 mM forskolin (MedChemExpress, Monmouth Junction, USA) or 0.1 mM 8-bromo-cAMP (MedChemExpress, Monmouth Junction, USA) was added to medium for 24 h.

### Sperm viability and sperm abnormality assessment

Fresh epididymides were obtained quickly after sacrifice. The caudae epididymis of each mouse was minced in 3 mL PBS and incubated at 37° C for 15 min. We used a hemocytometer to count the number of sperm in the supernatant and suspension. The viability of sperm was the ratio of the number of sperm in the supernatant to the number of sperm in the suspension. 10 μL suspension was dripped on a clean glass slide and the slides were dried. Then, the slides were stained with sperm staining solution following the manufacturer’s instructions (Huakang Biomedical Engineering, Shenzhen, China). The sperm morphology was observed by a DXM12000F microscope (Nikon, Tokyo, Japan). The person who was blinded to each group took pictures of the sperm and counted at least 1000 sperm. Abnormal sperm morphology manifested as acrosome loss, cephalic (small head), acephalia (no head), cervical folding, and tailless.

### H&E staining

The testis tissues were fixed with 4% paraformaldehyde for 4 h, dehydrated in a graded ethanol series and paraffin-embedded. The embedded testis tissues were sectioned at 5 μm. The slides were immersed in xylene for 10 min two times for dewaxing. Then, slides were dehydrated with an alcohol gradient concentration (100%, 95%, 90%, 70%, 50%, 30% alcohol and ddH_2_O for 3 min per wash). Dehydrated slides were stained with hematoxylin for 45 s, washed with water, and rinsed with ammonia. The slides were then immersed in an alcoholic solution of varying concentrations (30%, 50%, 70%, 90%) for 3 min, respectively. Subsequently, the slides were stained with 95% eosin for 5 min and soaked in 95% and 100% alcohol for 3 min. Finally, the slides were immersed in xylene and sealed with resin. Following staining, the sections were observed under a DXM12000F microscope (Nikon, Tokyo, Japan). Seminiferous tubules were selected for morphometric analysis. Tubule diameters (both longest and shortest) and germ cell layer thickness in at least 10 seminiferous tubules (round or nearly round) were analyzed from each slide of testicular cross-sections of control as well as experimental mice by using ImageJ.

### Quantitative real-time PCR (qRT-PCR)

Total RNA was extracted using Trizol reagent (Vazyme, Nanjing, China) according to the manufacturer’s protocol. Total RNA (1 μg) was reverse-transcribed into cDNA using an HiScript Q RT SuperMix (R123-01, Vazyme, Nanjing, China). The reaction conditions were 50° C for 15 min and 85° C for 2 min. QRT-PCR was performed using ChamQ Universal SYBR qPCR Master Mix (Q711-02, Vazyme, Nanjing, China) by Real Time PCR System (ABI Viia 7). The reaction conditions were 30 s at 95° C, followed by 45 cycles of denaturation at 95° C for 10 s, annealing at 60° C for 30 s, and extension at 72° C for 15 s. The primer sequences were shown in Additional file 1: Table S3. The relative quantification values of the target genes were measured by the 2^−△△Ct^ method, and GAPDH was used as an internal reference [51].

### Western blotting

Proteins were purified from testis tissues or Leydig cells by using RIPA buffer (Beyotime, Shanghai, China). The protein concentration was determined using BCA protein quantification kit (E112-02, Vazyme, Nanjing, China). Western blotting was carried out as previously mentioned [52]. Briefly, proteins were separated using 12% SDS–polyacrylamide gel electrophoresis and electrophoretically transferred to polyvinylidene fluoride (PVDF) membranes. The membrane was then blocked in PBS buffer containing 5% bovine serum albumin for 1 h at room temperature. Transferred blots were incubated with rabbit anti-CYP11A (P450scc) (Proteintech Group, Rosemont, IL, USA), rabbit anti-CYP17A (P450c17) (Proteintech Group, Rosemont, IL, USA), rabbit anti-StAR (Cell Signaling Technology, USA), mouse anti-HSD3β (Proteintech Group, Rosemont, IL, USA), rabbit anti-HSD17β2 (Proteintech Group, Rosemont, IL, USA), rabbit anti-LHR (Proteintech Group, Rosemont, IL, USA), and mouse anti-GAPDH (Proteintech Group, Rosemont, IL, USA) overnight at 4° C. Specific information on the antibodies was shown in Table 1. The transferred blots were incubated with the secondary antibody horseradish peroxidase-conjugated goat anti-rabbit/mouse IgG (Boster, Wuhan, China) for 1 h at room temperature. Protein signals were detected using ECL solution (E412-02, Vazyme, Nanjing, China) by gel imaging analysis system (Tanon 4200, Shanghai, China), and band intensities were quantified using ImageJ software (National Institutes of Health).

**Table 1:** Specifications of primary antibodies

Antibody	Species	Company	Catalog	Dilution
Anti-GAPDH	Mouse mAb	Proteintech	60004-1-lg	1:1000 (WB)
Anti-P450scc	Rabbit polyclonal Ab	Proteintech	13363-1-AP	1:1000 (WB)
Anti-P450c17	Rabbit polyclonal Ab	Proteintech	14447-1-AP	1:1000 (WB)
Anti-3β-HSD	Mouse mAb	Santa Cruz	sc-515120	1:1000 (WB)
Anti-17β-HSD	Rabbit polyclonal Ab	Proteintech	10978-1-AP	1:1000 (WB)
Anti-StAR	Rabbit mAb	Cell Signaling Technology	#8449	1:1000 (WB) 1:200 (IF)
Anti-LHR	Rabbit polyclonal Ab	Proteintech	19968-1-AP	1:1000 (WB)

### ELISA

The levels of testosterone, LH and FSH in serum were measured with ELISA kits (Elabscience Biotechnology Co., Ltd) according to the manufacturer's instructions. AC kinase activity, the content of cAMP, and PKA kinase activity were detected by ELISA kits (Jiangsu Meibiao Biotechnology Co., Ltd) according to the manufacturer's instructions. Intra- and inter-assay coefficients of variability, the detection limits, and specific information of ELISA kits were shown in Additional file 1: Table S4.

### Immunofluorescence staining

Testis tissues were fixed in 4% paraformaldehyde for 4 h at room temperature, dehydrated in a graded ethanol series and paraffin-embedded. The embedded testis tissues were sectioned at 5 μm. The slides were immersed in xylene for 10 min two times for dewaxing. Then slides were dehydrated with an alcohol gradient concentration (100%, 95%, 90%, 70%, 50%, and 30% alcohol for 3 min per wash). Triton (0.3%) was added to the glass slide for drilling for 10 min. Then the slides were washed with PBS for 10 min and sealed with 3% BSA solution at 37° C for 30 min. The sections were stained with the primary antibody rabbit anti-StAR (Cell Signaling Technology, USA), and incubated at 4° C overnight. After washing with PBS, the slides were incubated with the secondary antibody Alexa Fluor 594-conjugated goat anti-rabbit IgG at 37° C for 1 h. Nuclei were stained with DAPI (Sigma) at 37° C for 15 min. Images were captured using an FV10i microscope (Olympus, Japan).

### Immunohistochemistry

Paraffin-embedded testis tissue sections were soaked in xylene for 10 min two times. Then slides were dehydrated with an alcohol gradient concentration (100%, 95%, 90%, 70%, 50%, and 30% alcohol for 3 min per wash). The slides were incubated with PBS containing 3% H_2_O_2_ for 10 min to quench endogenous peroxidase activity. Triton (0.3%) was added to the glass slide for drilling for 10 min. After doing that, the slides were sealed at 37° C for 1 h in PBS containing 3% BSA and incubated with rabbit anti-LHR (Affinity, USA) at 4° C overnight. Whereafter, the slides were incubated in HRP-conjugated secondary antibodies (Boster, Wuhan, China) at 37° C for 1 h. Finally, we used the DAB Substrate System (DAKO) to observe the immunohistochemical staining under a DXM12000F microscope (Nikon, Tokyo, Japan).

### Statistical analysis

GraphPad Prism 8 (USA) was applied for statistical analysis. One-way analysis of variance (ANOVA) was used to analyze differences between groups, followed by Dunnett’s *t* test. The data were shown as means ± SD. The value *P* < 0.05 was regarded as statistically significant.

## Supplementary Information

**Additional file 1. Table S1.** Zeta potentials of PS-MPs. **Table S2.** Daily average food (g/day/mice) and water (mL/day/mice) consumption by male mice after exposure to PS-MPs. **Table S3.** Primer sequences used for qRT-PCR. **Table S4.** Specifications of ELISA kits. **Figure S1.** Effect of PS-MPs exposure on testicular morphometric parameters in mice. **Figure S2.** Effects of PS-MPs exposure on the number of Leydig cells in testes. **Figure S3.** Effects of PS-MPs exposure on the ROS level in Leydig cells. **Figure S4.** Effects of PS-MPs exposure on the testosterone level in the absence of Leydig cells. **Figure S5.** Identification of primary Leydig cells.

**Additional file 2.** Cell imaging.

## Data Availability

The datasets supporting the conclusions of this article are included within the article, Supplementary Materials, and cell imaging.

## References

[1] Moore CJ (2008). Synthetic polymers in the marine environment: a rapidly increasing, long-term threat. Environ Res.

[2] Rochman CM, Browne MA, Halpern BS, Hentschel BT, Hoh E, Karapanagioti HK (2013). Policy: classify plastic waste as hazardous. Nature.

[3] Cozar A, Echevarria F, Gonzalez-Gordillo JI, Irigoien X, Ubeda B, Hernandez-Leon S (2014). Plastic debris in the open ocean. Proc Natl Acad Sci USA.

[4] Andrady AL (2011). Microplastics in the marine environment. Mar Pollut Bull.

[5] Thompson RC, Olsen Y, Mitchell RP, Davis A, Rowland SJ, John AW (2004). Lost at sea: where is all the plastic?. Science.

[6] Barnes DK, Galgani F, Thompson RC, Barlaz M (2009). Accumulation and fragmentation of plastic debris in global environments. Philos Trans R Soc Lond B Biol Sci.

[7] Steer M, Cole M, Thompson RC, Lindeque PK (2017). Microplastic ingestion in fish larvae in the western English Channel. Environ Pollut.

[8] Eerkes-Medrano D, Thompson RC, Aldridge DC (2015). Microplastics in freshwater systems: a review of the emerging threats, identification of knowledge gaps and prioritisation of research needs. Water Res.

[9] Wu P, Huang J, Zheng Y, Yang Y, Zhang Y, He F (2019). Environmental occurrences, fate, and impacts of microplastics. Ecotoxicol Environ Saf.

[10] Eriksen M, Mason S, Wilson S, Box C, Zellers A, Edwards W (2013). Microplastic pollution in the surface waters of the Laurentian Great Lakes. Mar Pollut Bull.

[11] Rillig MC (2012). Microplastic in terrestrial ecosystems and the soil?. Environ Sci Technol.

[12] Wagner M, Scherer C, Alvarez-Munoz D, Brennholt N, Bourrain X, Buchinger S (2014). Microplastics in freshwater ecosystems: what we know and what we need to know. Environ Sci Eur.

[13] Sadri SS, Thompson RC (2014). On the quantity and composition of floating plastic debris entering and leaving the Tamar Estuary, Southwest England. Mar Pollut Bull.

[14] Miranda DA, de Carvalho-Souza GF (2016). Are we eating plastic-ingesting fish?. Mar Pollut Bull.

[15] Setala O, Fleming-Lehtinen V, Lehtiniemi M (2014). Ingestion and transfer of microplastics in the planktonic food web. Environ Pollut.

[16] Yang YF, Chen CY, Lu TH, Liao CM (2019). Toxicity-based toxicokinetic/toxicodynamic assessment for bioaccumulation of polystyrene microplastics in mice. J Hazard Mater.

[17] Dong CD, Chen CW, Chen YC, Chen HH, Lee JS, Lin CH (2020). Polystyrene microplastic particles: in vitro pulmonary toxicity assessment. J Hazard Mater.

[18] Umamaheswari S, Priyadarshinee S, Bhattacharjee M, Kadirvelu K, Ramesh M (2020). Exposure to polystyrene microplastics induced gene modulated biological responses in zebrafish (*Danio rerio*). Chemosphere.

[19] Lu Y, Zhang Y, Deng Y, Jiang W, Zhao Y, Geng J (2016). Uptake and accumulation of polystyrene microplastics in Zebrafish (*Danio rerio*) and toxic effects in liver. Environ Sci Technol.

[20] Deng Y, Zhang Y, Lemos B, Ren H (2017). Tissue accumulation of microplastics in mice and biomarker responses suggest widespread health risks of exposure. Sci Rep.

[21] Ding J, Zhang S, Razanajatovo RM, Zou H, Zhu W (2018). Accumulation, tissue distribution, and biochemical effects of polystyrene microplastics in the freshwater fish red tilapia (*Oreochromis niloticus*). Environ Pollut.

[22] Zheng H, Wang J, Wei X, Chang L, Liu S (2021). Proinflammatory properties and lipid disturbance of polystyrene microplastics in the livers of mice with acute colitis. Sci Total Environ.

[23] Jin Y, Lu L, Tu W, Luo T, Fu Z (2019). Impacts of polystyrene microplastic on the gut barrier, microbiota and metabolism of mice. Sci Total Environ.

[24] Barboza LGA, Vieira LR, Branco V, Figueiredo N, Carvalho F, Carvalho C (2018). Microplastics cause neurotoxicity, oxidative damage and energy-related changes and interact with the bioaccumulation of mercury in the *European seabass*, *Dicentrarchus labrax* (Linnaeus, 1758). Aquat Toxicol.

[25] Murphy F, Quinn B (2018). The effects of microplastic on freshwater *Hydra attenuata* feeding, morphology & reproduction. Environ Pollut.

[26] Assas M, Qiu X, Chen K, Ogawa H, Xu H, Shimasaki Y (2020). Bioaccumulation and reproductive effects of fluorescent microplastics in medaka fish. Mar Pollut Bull.

[27] Sussarellu R, Suquet M, Thomas Y, Lambert C, Fabioux C, Pernet ME (2016). Oyster reproduction is affected by exposure to polystyrene microplastics. Proc Natl Acad Sci USA.

[28] Hou B, Wang F, Liu T, Wang Z (2021). Reproductive toxicity of polystyrene microplastics: in vivo experimental study on testicular toxicity in mice. J Hazard Mater.

[29] Xie X, Deng T, Duan J, Xie J, Yuan J, Chen M (2020). Exposure to polystyrene microplastics causes reproductive toxicity through oxidative stress and activation of the p38 MAPK signaling pathway. Ecotoxicol Environ Saf.

[30] Amereh F, Babaei M, Eslami A, Fazelipour S, Rafiee M (2020). The emerging risk of exposure to nano(micro)plastics on endocrine disturbance and reproductive toxicity: from a hypothetical scenario to a global public health challenge. Environ Pollut.

[31] Zirkin BR, Papadopoulos V (2018). Leydig cells: formation, function, and regulation. Biol Reprod.

[32] Smith LB, Walker WH (2014). The regulation of spermatogenesis by androgens. Semin Cell Dev Biol.

[33] Dufau ML (1988). Endocrine regulation and communicating functions of the Leydig cell. Annu Rev Physiol.

[34] Tremblay JJ (2015). Molecular regulation of steroidogenesis in endocrine Leydig cells. Steroids.

[35] Jeong CB, Won EJ, Kang HM, Lee MC, Hwang DS, Hwang UK (2016). Microplastic size-dependent toxicity, oxidative stress induction, and p-JNK and p-p38 activation in the Monogonont Rotifer (*Brachionus koreanus*). Environ Sci Technol.

[36] Yu P, Liu Z, Wu D, Chen M, Lv W, Zhao Y (2018). Accumulation of polystyrene microplastics in juvenile *Eriocheir sinensis* and oxidative stress effects in the liver. Aquat Toxicol.

[37] D'Angelo S, Meccariello R (2021). Microplastics: a threat for male fertility. Int J Environ Res Public Health.

[38] Lu L, Wan Z, Luo T, Fu Z, Jin Y (2018). Polystyrene microplastics induce gut microbiota dysbiosis and hepatic lipid metabolism disorder in mice. Sci Total Environ.

[39] Yan HH, Mruk DD, Lee WM, Cheng CY (2008). Blood-testis barrier dynamics are regulated by testosterone and cytokines via their differential effects on the kinetics of protein endocytosis and recycling in Sertoli cells. FASEB J.

[40] Meng J, Holdcraft RW, Shima JE, Griswold MD, Braun RE (2005). Androgens regulate the permeability of the blood-testis barrier. Proc Natl Acad Sci USA.

[41] Su L, Mruk DD, Lee WM, Cheng CY (2010). Differential effects of testosterone and TGF-beta3 on endocytic vesicle-mediated protein trafficking events at the blood-testis barrier. Exp Cell Res.

[42] Yeh S, Tsai MY, Xu Q, Mu XM, Lardy H, Huang KE (2002). Generation and characterization of androgen receptor knockout (ARKO) mice: an in vivo model for the study of androgen functions in selective tissues. Proc Natl Acad Sci USA.

[43] O'Donnell L, McLachlan RI, Wreford NG, de Kretser DM, Robertson DM (1996). Testosterone withdrawal promotes stage-specific detachment of round spermatids from the rat seminiferous epithelium. Biol Reprod.

[44] Holdcraft RW, Braun RE (2004). Androgen receptor function is required in Sertoli cells for the terminal differentiation of haploid spermatids. Development.

[45] Jin H, Ma T, Sha X, Liu Z, Zhou Y, Meng X (2021). Polystyrene microplastics induced male reproductive toxicity in mice. J Hazard Mater.

[46] Ramsperger A, Narayana VKB, Gross W, Mohanraj J, Thelakkat M, Greiner A (2020). Environmental exposure enhances the internalization of microplastic particles into cells. Sci Adv.

[47] Manna PR, Dyson MT, Eubank DW, Clark BJ, Lalli E, Sassone-Corsi P (2002). Regulation of steroidogenesis and the steroidogenic acute regulatory protein by a member of the cAMP response-element binding protein family. Mol Endocrinol.

[48] Nishimura R, Shibaya M, Skarzynski DJ, Okuda K (2004). Progesterone stimulation by LH involves the phospholipase-C pathway in bovine luteal cells. J Reprod Dev.

[49] Liu MY, Lai HY, Yang BC, Tsai ML, Yang HY, Huang BM (2001). The inhibitory effects of lead on steroidogenesis in MA-10 mouse Leydig tumor cells. Life Sci.

[50] Ma W, Li S, Ma S, Jia L, Zhang F, Zhang Y (2016). Zika virus causes testis damage and leads to male infertility in mice. Cell.

[51] Livak KJ, Schmittgen TD (2001). Analysis of relative gene expression data using real-time quantitative PCR and the 2(-Delta Delta C(T)) method. Methods.

[52] Ding J, Wang J, Jin H, Xia T, Cheng Y, Wu J (2018). Microcystin-LR reduces the synthesis of gonadotropin-releasing hormone by activating multiple signaling pathways resulting in decrease of testosterone in mice. Sci Total Environ.

