# Supplementary material for: Chronic exposure to polystyrene microplastics induced male reproductive toxicity and decreased testosterone levels via the LH-mediated LHR/cAMP/PKA/StAR pathway
Source: Part Fibre Toxicol. 2022 Feb 17;19:13. doi: 10.1186/s12989-022-00453-2 (PMC8851716; doi:10.1186/s12989-022-00453-2)
Supplement: Supplementary file 1 — Additional file 1. Table S1. Zeta potentials of PS-MPs. Table S2. Daily average food (g/day/mice) and water (mL/day/mice) consumption by male mice after exposure to PS-MPs. Table S3. Primer sequences used for qRT-PCR. Table S4. Specifications of ELISA kits. Figure S1. Effect of PS-MPs exposure on testicular morphometric parameters in mice. Figure S2. Effects of PS-MPs exposure on the number of Leydig cells in testes. Figure S3. Effects of PS-MPs exposure on the ROS level in Leydig cells. Figure S4. Effects of PS-MPs exposure on the testosterone level in the absence of Leydig cells. Figure S5. Identification of primary Leydig cells. [file 12989_2022_453_MOESM1_ESM.docx]

**Chronic exposure to polystyrene microplastics induced male reproductive toxicity and decreased testosterone levels via the LH-mediated LHR/cAMP/PKA/StAR pathway**

Haibo Jin^1, 2^, Minghao Yan^1, 2^, Chun Pan^1, 2^, Zhenyu Liu^1, 2^, Xiaoxuan Sha^1, 2^, Chengyue Jiang^1, 2^, Luxi Li^1, 2^, Mengge Pan^1, 2^, Dongmei Li^1, 2^, Xiaodong Han^1, 2, *^, Jie Ding^1, 2, *^

1 Immunology and Reproductive Biology Laboratory & State Key Laboratory of Analytical Chemistry for Life Science, Medical School, Nanjing University, Hankou Road 22, Nanjing, Jiangsu 210093, China;
2 Jiangsu Key Laboratory of Molecular Medicine, Nanjing University, Nanjing 210093, China;
* Corresponding author at: Immunology and Reproduction Biology Laboratory & State Key Laboratory of Analytical Chemistry for Life Science, Medical School, Nanjing University, Nanjing, Jiangsu 210093, China.

1. mail address: [djie@nju.edu.cn](mailto:djie@nju.edu.cn) (J. Ding); [hanxd@nju.edu.cn](mailto:hanxd@nju.edu.cn) (X. Han); [jin1144827403@163.com](mailto:jin1144827403@163.com) (H. Jin); [ymh__66@163.com](mailto:ymh__66@163.com) (M. Yan); [panchun0211@163.com](mailto:panchun0211@163.com) (C. Pan); [LIUZY20000424@yeah.net](mailto:LIUZY20000424@yeah.net) (Z. Liu); [171230016@smail.nju.edu.cn](mailto:171230016@smail.nju.edu.cn) (X. Sha); [jcyue@163.com](mailto:jcyue@163.com) (C. Jiang); [lucysenior2014@163.com](mailto:lucysenior2014@163.com) (L. Li); [pmgenjoy2018@outlook.com](mailto:pmgenjoy2018@outlook.com) (M. Pan); [lidm@nju.edu.cn](mailto:lidm@nju.edu.cn) (D. Li)

**Table S1.** Zeta potentials of PS-MPs.

| 0.5 μm | 4 μm | 10 μm |
| --- | --- | --- |
| -39.63 mV | -29.23 mV | -39.62 mV |
| -49.27 mV | -32.97 mV | -34.34 mV |
| -44.25 mV | -32.95 mV | -35.07 mV |

**Table S2.** Daily average food (g/day/mice) and water (mL/day/mice) consumption by male mice after exposure to PS-MPs.

| group | Control | 0.5 μm  (100 μg/L) | 0.5 μm  (1000 μg/L) | 4 μm  (100 μg/L) | 4 μm  (1000 μg/L) | 10 μm  (100 μg/L) | 10 μm  (1000 μg/L) |
| --- | --- | --- | --- | --- | --- | --- | --- |
| Food Intake | 4.1 ± 0.3 | 3.5 ± 0.2 | 3.2 ± 0.3 | 3.4 ± 0.4 | 3.2 ± 0.2 | 3.4 ± 0.5 | 3.1 ± 0.3 |
| Water Intake | 3.1 ± 0.2 | 3.2 ± 0.1 | 3.2 ± 0.3 | 3.3 ± 0.4 | 3.1 ± 0.3 | 3.2 ± 0.4 | 3.1 ± 0.5 |
| Body Weight | 28.06 ± 0.83 | 27.18 ± 2.46 | 25.84 ± 4.83 | 28.11 ± 0.8 | 26.26 ± 2.68 | 27.65 ± 2.14 | 26.22 ± 3.7 |

**Table S3.** Primer sequences used for qRT-PCR.

| Gene | Primer | Sequence 5′ → 3′ | Product size (base pairs) |
| --- | --- | --- | --- |
| GAPDH | Forward | AGGTCGGTGTGAACGGATTTG | 178 |
|  | Reverse | TGTAGACCATGTAGTTGAGTCA |  |
| P450scc | Forward | CTTTGGTGCAGGTGGCTAG | 115 |
|  | Reverse | CGGAAGTGCGTGGTGTTT |  |
| P450c17 | Forward | GCCCAAGTCAAAGACACCTAAT | 159 |
|  | Reverse | GTACCCAGGCGAAGAGAATAGA |  |
| 3β-HSD | Forward | TGTGCCAGCCTTCATCTAC | 145 |
|  | Reverse | CTTCTCGGCCATCCTTTT |  |
| 17β-HSD | Forward | ACTTGGCTGTTCGCCTAGC | 117 |
|  | Reverse | GAGGGCATCCTTGAGTCCTG |  |
| StAR | Forward | ATGTTCCTCGCTACGTTCAAG | 122 |
|  | Reverse | CCCAGTGCTCTCCAGTTGAG |  |
| LHR | Forward | CTCGCCCGACTATCTCTCAC | 77 |
|  | Reverse | ACGACCTCATTAAGTCCCCTG |  |

**Table S4.** Specifications of ELISA kits.

|  | Company | Catalog | Detection Range | Intra-assay coefficient of variability (%) | Inter-assay coefficient of variability (%) |
| --- | --- | --- | --- | --- | --- |
| Testosterone | Elabscience Biotechnology | E-EL-0155c | 0.31-20 ng/mL | 4.2 | 4.7 |
| FSH | Elabscience Biotechnology | E-EL-M0511c | 1.56-100 ng/mL | 5.0 | 6.3 |
| LH | Elabscience Biotechnology | E-EL-M3053c | 0.31-20 ng/mL | 4.6 | 7.1 |
| cAMP | Jiangsu Meibiao Biotechnology | MB-3280A | 0.375-12 pmol/mL | 4.7 | 6.5 |
| PKA | Jiangsu Meibiao Biotechnology | MB-5746A | 15-480 U/L | 5.2 | 5.6 |
| AC | Jiangsu Meibiao Biotechnology | MB-5970A | 25-800 U/L | 6.6 | 6.9 |


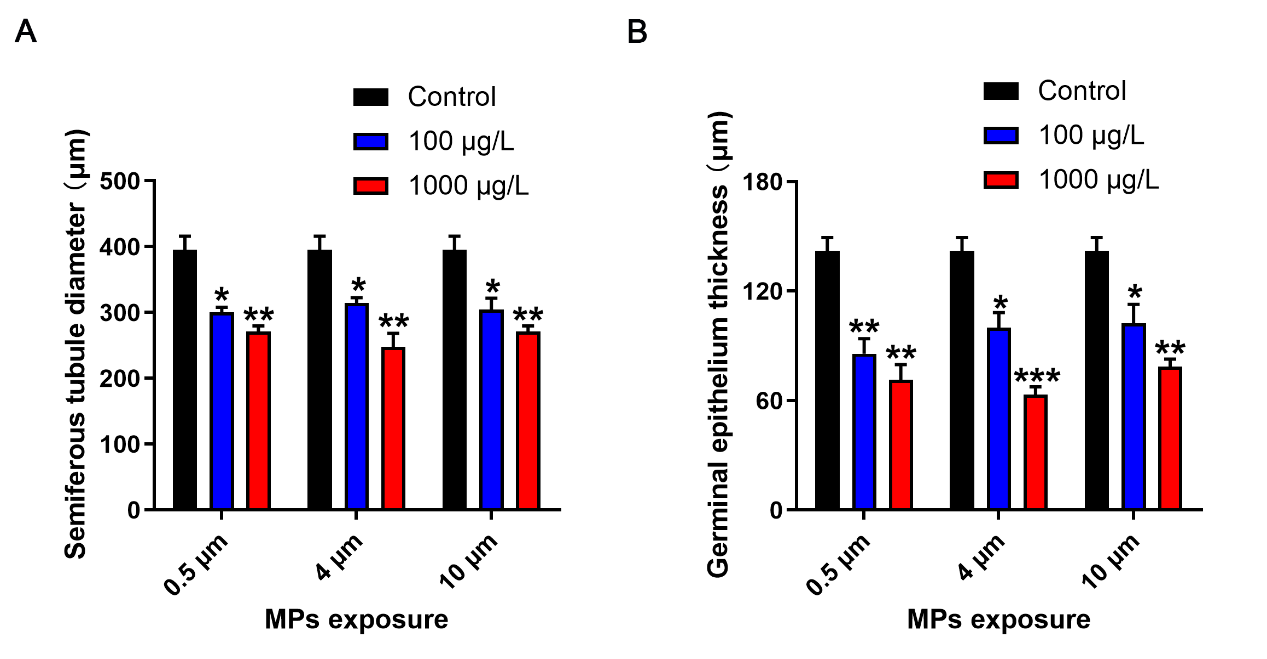


**Figure S1.** Effect of PS-MPs exposure on testicular morphometric parameters in mice. (A) Seminiferous tubular diameter. (B) Germinal cell thickness. Data are expressed as means ± SD. **P* < 0.05, ***P* < 0.01, ****P* < 0.001 vs. control.


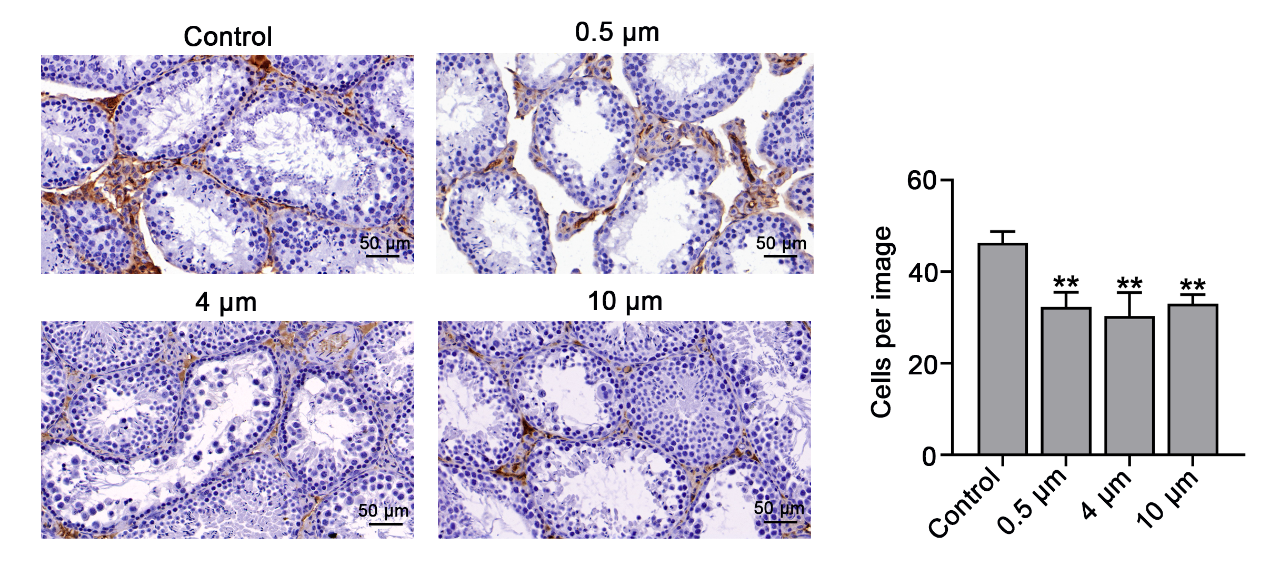


**Figure S2.** Effects of PS-MPs exposure on the number of Leydig cells in testes. Mice were given drinking water containing various particle sizes of PS-MPs as indicated for 180 consecutive days. Immunohistochemical staining for 3βHSD were performed (scale bar = 50 μm). Each testis was analyzed based on four sections, with 1000 cells counted per section. Percent of positivity was calculated based on the percentage of 3βHSD positive cells out of the total number of cells in an image (n = 8 mice/group). Data are presented as the means ± SD. ***P* < 0.01, compared with the control.


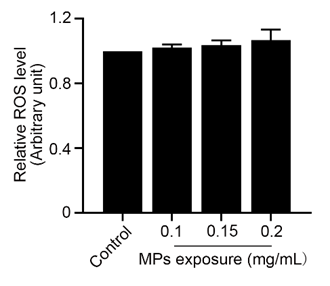


**Figure S3.** Effects of PS-MPs exposure on the ROS level in Leydig cells. Primary Leydig cells were exposed to 0.5 μm PS-MPs for 24 h at various concentrations as indicated. The DCF fluorescence intensity was detected DCFH-DA staining and measured using a microplate reader.


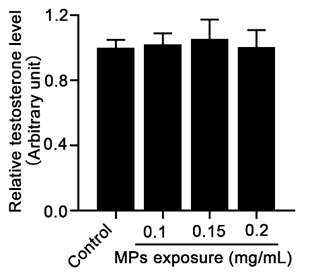


**Figure S4.** Effects of PS-MPs exposure on the testosterone level in the absence of Leydig cells. Different concentrations of PS-MPs were added to the culture medium with the same concentration of testosterone in the absence of Leydig cells. After 24 h, the testosterone contents in the culture medium were detected by ELISA.


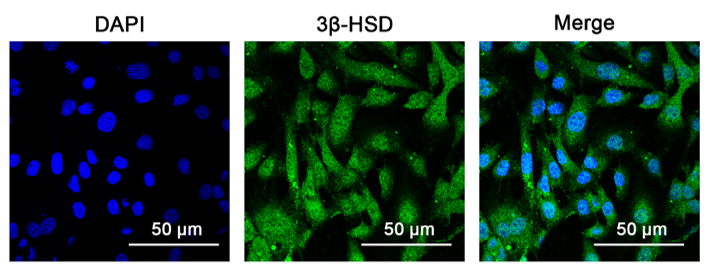


**Fig. S5.** Identification of primary Leydig cells. Cells were stained with 3βHSD (green) and DAPI (blue) (scale bar = 50 μm).
